# Pathway-Dependent Regulation of Sleep Dynamics in a Network Model of the Sleep–Wake Cycle

**DOI:** 10.3389/fnins.2019.01380

**Published:** 2019-12-20

**Authors:** Charlotte Héricé, Shuzo Sakata

**Affiliations:** Strathclyde Institute of Pharmacy and Biomedical Sciences, University of Strathclyde, Glasgow, United Kingdom

**Keywords:** sleep regulatory circuits, computational model, brain state, sleep/wake cycle, Python programing language

## Abstract

Sleep is a fundamental homeostatic process within the animal kingdom. Although various brain areas and cell types are involved in the regulation of the sleep–wake cycle, it is still unclear how different pathways between neural populations contribute to its regulation. Here we address this issue by investigating the behavior of a simplified network model upon synaptic weight manipulations. Our model consists of three neural populations connected by excitatory and inhibitory synapses. Activity in each population is described by a firing-rate model, which determines the state of the network. Namely wakefulness, rapid eye movement (REM) sleep or non-REM (NREM) sleep. By systematically manipulating the synaptic weight of every pathway, we show that even this simplified model exhibits non-trivial behaviors: for example, the wake-promoting population contributes not just to the induction and maintenance of wakefulness, but also to sleep induction. Although a recurrent excitatory connection of the REM-promoting population is essential for REM sleep genesis, this recurrent connection does not necessarily contribute to the maintenance of REM sleep. The duration of NREM sleep can be shortened or extended by changes in the synaptic strength of the pathways from the NREM-promoting population. In some cases, there is an optimal range of synaptic strengths that affect a particular state, implying that the amount of manipulations, not just direction (i.e., activation or inactivation), needs to be taken into account. These results demonstrate pathway-dependent regulation of sleep dynamics and highlight the importance of systems-level quantitative approaches for sleep–wake regulatory circuits.

## Introduction

Global brain states vary dynamically on multiple timescales. Humans typically exhibit a daily cycle between three major behavioral states: wakefulness, REM sleep and NREM sleep. This daily cycle is regulated by a circadian rhythm and a homeostatic sleep pressure (Borbély, 1982; Achermann and Borbely, 1990). These states alternate on a timescale of several hours called an ultradian rhythm (Borbély, 1982; Archermann and Borbely, 2017; Carskadon, 2017). Thus, complex interactions between homeostatic, circadian, and ultradian processes are involved in the sleep-wake cycle generation. However, it remains elusive how these states are regulated in the brain.

Over the past several decades, various cell types, neurotransmitters and neuropeptides have been identified as part of the sleep–wake regulating circuits within the brain (Saper et al., 2001; Brown et al., 2012; Luppi et al., 2013; Weber and Dan, 2016; Scammell et al., 2017; Herice et al., 2019). Sleep- or wake-promoting neurons show state-dependent firing and contribute to the induction and/or maintenance of a particular state (Jouvet, 1962; McCarley and Hobson, 1971; Hobson et al., 1975; Saper et al., 2001; Brown et al., 2012; Weber and Dan, 2016; Herice et al., 2019). To gain a better understanding of sleep–wake regulation, it is fundamental not just to identify and characterize each component of sleep–wake regulating circuits, but to also investigate how each pathway between neural populations contributes to state regulation.

Although controlling neural activity has provided mechanistic insights into sleep–wake regulation, their results are sometimes contradictory: for example, the role of pontine cholinergic neurons in REM sleep has been debated (Grace et al., 2014; Grace, 2015; Grace and Horner, 2015; Van Dort et al., 2015). Even recent studies with opto- and chemogenetic approaches do not resolve this long-standing issue (Van Dort et al., 2015; Kroeger et al., 2017). Even though this discrepancy may be simply due to differences in animal models and experimental techniques, it is technically challenging to manipulate neurons or specific pathways precisely across different laboratories.

A computational approach may be a viable alternative for gaining insights into the mechanism of sleep–wake regulation. Since pioneering work in the 1970s and 1980s (McCarley and Hobson, 1975; Borbély, 1982; Archermann and Borbely, 2017), various computational models have been developed (Tamakawa et al., 2006; Diniz Behn et al., 2007; Diniz-Behn and Booth, 2010; Robinson et al., 2011; Booth and Diniz Behn, 2014; Archermann and Borbely, 2017; Booth et al., 2017; Herice et al., 2019): conceptually, a homeostatic sleep-dependent process and a circadian process play a key role in sleep regulation (Borbély, 1982; Archermann and Borbely, 2017). Reciprocal excitatory-inhibitory connections (McCarley and Hobson, 1975; Diniz Behn et al., 2007; Diniz-Behn and Booth, 2010; Diniz Behn and Booth, 2012; Booth et al., 2017) and mutual inhibitory interactions (Saper et al., 2001) can be recognized as key network motifs within sleep–wake regulating circuits. Although their dynamics have been explored (Diniz Behn and Booth, 2012; Diniz Behn et al., 2013; Weber, 2017), and those models can replicate sleep architecture of humans and animals (Diniz-Behn and Booth, 2010) as well as state-dependent neural firing (Tamakawa et al., 2006), few studies have investigated how the strength of synaptic connections between wake- and sleep-promoting populations contribute to sleep dynamics. As controlling neural activity at high spatiotemporal resolution *in vivo* becomes feasible experimentally, computational approaches can be considered as complementary approaches for investigating the role of specific neural pathways in sleep–wake regulation.

**FIGURE 1:**
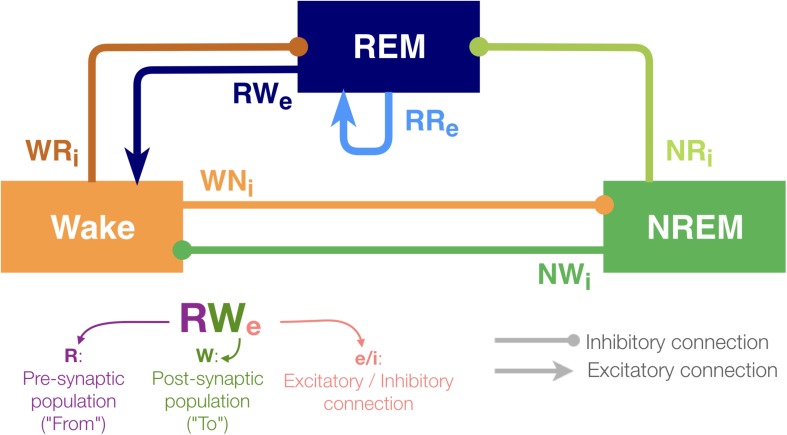
Architecture of the sleep regulatory network. Three neural populations are connected with excitatory and inhibitory synapses. Each neural population is named as the state they promote. The arrows and circles represent excitatory and inhibitory connections, respectively. The synapses are named with two uppercase and one lowercase letters: first letter of the pre-synaptic population (where the synapse is from), first letter of the post-synaptic population (where the synapse is going to) and “e” if it is excitatory or sign “i” if inhibitory.

To this end, we utilize a simplified network model (Diniz Behn and Booth, 2012; Costa et al., 2016) (Figure 1) and systematically manipulate the strength of every pathway. Because neurons within the sleep–wake regulating circuits typically project to a wide range of neural populations (Schwarz and Luo, 2015; Scammell et al., 2017; Herice et al., 2019), their contributions to the sleep–wake cycle may also vary depending on the pathway. Therefore, we set out to test the hypothesis that the sleep–wake cycle is regulated in a pathway-dependent manner.

Although the present model is highly abstract, it captures the following key features of sleep–wake regulating circuits: while the interaction between neuronal populations in the brainstem and the hypothalamus governs the sleep–wake regulation, some of the populations can be recognized as wake- or sleep-promoting (Brown et al., 2012; Luppi et al., 2013, 2017; Scammell et al., 2017; Herice et al., 2019). To reflect the populations’ state-dependent firing, the model contains three neuronal populations (REM, NREM and Wake). The activity in these populations defines the state of the network (see section Materials and Methods).

With respect to connectivity between these populations, Saper et al. (2001) proposed that the mutual inhibition between wake-promoting and sleep-promoting populations acts as a flip-flop switch for the regulation of transitioning between wakefulness and NREM sleep. Hence, in this model, the outputs from the Wake-promoting and NREM-promoting populations are considered as inhibitory. Because pontine REM-active cholinergic neurons provide excitatory connections to the sublaterodorsal nucleus (SLD), a key component of REM sleep-regulating circuits (Boissard et al., 2002), the REM-promoting population has a recurrent excitatory connection. Glutamatergic neurons project rostrally to several wake-promoting nuclei, such as the intralaminar nuclei of the thalamus and basal forebrain, and the REM population also provides excitatory outputs onto the Wake population (Boissard et al., 2002; Lu et al., 2006). In addition, because recent studies have shown that GABAergic inputs play a role in REM sleep induction (Weber et al., 2015), the REM-promoting population also receives inhibitory inputs from both the wake-promoting and NREM-promoting populations in this model. Based on this simplified model, we report that the effects of synaptic weight alterations on sleep architecture are highly pathway-dependent. We also discuss implications for future biological experiments.

## Materials and Methods

We implemented a computational model of the sleep/wake cycle containing three neuronal populations whose activity by several differential equations. Numerical simulations were computed with the Runge–Kutta integration method (4th order), with a time step of 1 ms and a simulation duration of 24 h. For these simulations and a part of the data processing, we used the Python programing language (version 3.6.8). In order to run multiple simulations for all the conditions, we implemented a script Bash (version 3.2.57). The majority of the data processing, the plots were performed with R (version 3.5.1) and MATLAB (R2018b, Mathworks). All details about the tools and libraries used for this work are summarized in Supplementary Table S1. Codes are available at https://github.com/Sakata-Lab/sleep-model.

### Firing Rate Formalism

All three populations are promoting the sleep–wake cycle corresponding to their name and are associated with a specific neurotransmitter. The REM-promoting population releases the excitatory neurotransmitters RX_*e*_ whereas the NREM- and Wake-promoting populations release the inhibitory ones NX_*i*_ and WX_*i*_, respectively.

Firing rate *F*_*X*_ of population *X* is described as follows:

$$\frac{d\,F_{X}}{d\,t}=\frac{F_{X\,\infty }\,\left(I_{X}\right)-F_{X}}{\tau_{X}},$$

where *F*_*X∞*_ is a steady state firing rate function for each population (see below). *τ*_*X*_ is the membrane time constant of the population *X*. The synaptic input *I*_*X*_ is a weighted sum of neurotransmitter concentrations released by the pre-synaptic populations *Y* and is described as follows:

$$I_{W}=g_{N\,W_{i}}\cdot C_{N\,X_{i}}+g_{R\,W_{e}}\cdot C_{R\,X_{e}}+\xi \,\left(t\right)$$

$$I_{N}=g_{W\,N_{i}}\cdot C_{W\,X_{i}}+\xi \,\left(t\right)$$

$$I_{R}=g_{W\,R_{i}}\cdot C_{W\,X_{i}}+g_{N\,R_{i}}\cdot C_{N\,X_{i}}+g_{R\,R_{e}}\cdot C_{R\,X_{e}}+\xi \,\left(t\right),$$

where *C*_*YXe/i*_ represents the neurotransmitter concentration involved in the pathway from population *Y* to *X* with synaptic weight *g*_*YXe/i*_. The parameter *ξ*(*t*) provides a weak Gaussian noise (mean of 0.01 Hz and standard deviation of 0.005 Hz) to mimics the variability of the biological networks.

For each population, the steady state firing rate function *F*_*X∞*_ is modeled with the following equations:

$$F_{W\,\infty }=W_{\max}\cdot \left(0.5\cdot \left(1+\tanh\left[\left(I_{W}-\beta_{W}\right)/\alpha_{W}\right]\right)\right)$$

$$F_{R\,\infty }=R_{\max}\cdot \left(0.5\cdot \left(1+\tanh\left[\left(I_{R}-\beta_{R}\right)/\alpha_{R}\right]\right)\right)$$

$$F_{N\,\infty }=N_{\max}\cdot \left(0.5\cdot \left(1+\tanh\left[\left(I_{N}-k_{N}\cdot H\,\left(t\right)\right)/\alpha_{N}\right]\right)\right),$$

where W_*max*_, N_*max*_ and R_*max*_ are constant values to set the maximum firing rates of the populations. α and β are slope and threshold parameters of the hyperbolic tangent function for the population X, respectively. Because the NREM population is linked to the homeostatic sleep drive, the steady state firing function also depends on the homeostatic sleep drive variable *H*(*t*) (see below).

All parameter values are provided in Supplementary Table S2.

### Homeostatic Sleep Drive

In the model, the sleep–wake transition is driven by the homeostatic sleep drive *H*(*t*). This process can be described by the following equation:

$$\frac{d\,H}{d\,t}=\frac{H_{\max}-H}{\tau_{h\,w}}\cdot ℋ\,\left(F_{W}-\theta_{W}\right)-\frac{H}{\tau_{h\,s}}\cdot ℋ\,\left(\theta_{W}-F_{W}\right),$$

where ℋ(*z*) stands for the Heaviside function, which returns 0 if *z* < 0 and 1 if z ≥ 0. θ*_*W*_* is a constant to set the sleep drive threshold. *H*_*max*_ is a constant value to set the maximum value for the homeostatic force. T*_*hw*_* and T*_*hs*_* are time constants of sleep drive built up during wakefulness and declined during sleep, respectively. Hence, the homeostatic force value increases during wakefulness due to a high activity of the wake-promoting population, and decreases during sleep when this activity is lower.

### Action of Neurotransmitters

The neurotransmitter concentration *C_*YX*_(t)* from the populations Y to X is described as following:

$$\frac{d\,C_{Y\,X}}{d\,t}=\frac{C_{Y\,X_{\infty }}\,\left(F_{Y}\right)-C_{Y\,X}}{\tau_{Y\,X}},$$

where *C_*YX*__8_* is a saturating function to provide the steady state of the neurotransmitter release from the population Y to the population X as a function of F_*Y*_. This function is described as:

$$C_{Y\,X_{\infty }}=\tanh\left(F_{Y}/\tau_{Y\,X}\right),$$

where T*_*YX*_* is a time constant. The concentration of each neurotransmitter was normalized between 0 and 1 and is expressed in arbitrary unit (a.u.) (Diniz-Behn and Booth, 2010).

### Alterations of Synaptic Weights in the Network

To investigate pathway-dependent regulation of sleep architecture in the network model, we systematically altered the synaptic weight *g* in the network as shown in Table 1.

**TABLE 1:** Synaptic weights for the different alterations.

**Conditions**	**Eighth**	**Quarter**	**Half**	**Double**	**Quadruple**	**Octuple**
**Symbols**	**g/8**	**g/4**	**g/2**	**g^∗^2**	**g^∗^4**	**g^∗^8**
RR_*e*_	0.2	0.4	0.8	3.2	6.4	12.8
RW_*e*_	0.125	0.25	0.5	2.0	4.0	8.0
WN_*i*_	–0.25	–0.5	–1.0	–4.0	–8.0	–16.0
WR_*i*_	–0.5	–1.0	–2.0	–8.0	–16.0	–32.0
NR_*i*_	–0.1625	–0.325	–0.65	–2.6	–5.2	–10.4
NW_*i*_	–0.21	–0.42	–0.84	–3.36	–6.72	–13.44

*Initials values can be found in the Supplementary Table S2 with the model parameters.*

We also simulated a lesion of each pathway by setting *g* to 0. For each condition, we run 8 simulations.

### Determination of Sleep–Wake States

The state of the network was determined according to Diniz-Behn and Booth (2010): If firing rate of the Wake-promoting population is above 2 Hz, the state of the network is Wake. If not, the state is either NREM or REM sleep: if firing rate of the REM-promoting population is above 2 Hz, the state is REM sleep. If not, the state is NREM sleep.

### Statistical Analyses

All statistical analyses were performed using R scripts (version 3.5.1). Data are presented as the means (plain curves) ± s.e.m. (shaded curves). One-way analysis of variance (ANOVA) were used to analyze the synaptic weights alterations depending on the sleep state or transition. Following the ANOVA, Tukey *post hoc* tests were performed for pairwise comparisons to the control conditions (no synaptic weights manipulations). *P*-values less than 0.05 were considered significant. If it is not the case, the sign “NS” was added on the graphs, otherwise there was a significant difference compared to the control condition.

## Results

We utilized the network architecture as reported in previous studies (Diniz Behn and Booth, 2012; Costa et al., 2016). As shown in Figure 1, this model contained three neuronal populations (labeled REM, NREM and Wake). The activity of these populations was characterized by differential equations describing the population firing rates which defined the state of the network (see Materials and Methods). These equations have been proved to be components of suitable sleep/wake regulatory computational models in previous studies (Diniz Behn et al., 2007, 2013; Diniz-Behn and Booth, 2010; Diniz Behn and Booth, 2012; Costa et al., 2016). The pathways from one population to the other were either excitatory or inhibitory. The concentrations of excitatory and inhibitory neurotransmitters were directly related to the population firing rates of the neural populations and a homeostatic sleep drive. Each population also received random Gaussian noise (Supplementary Figure S1).

### Sleep Dynamics Under Initial Conditions

**FIGURE 2:**
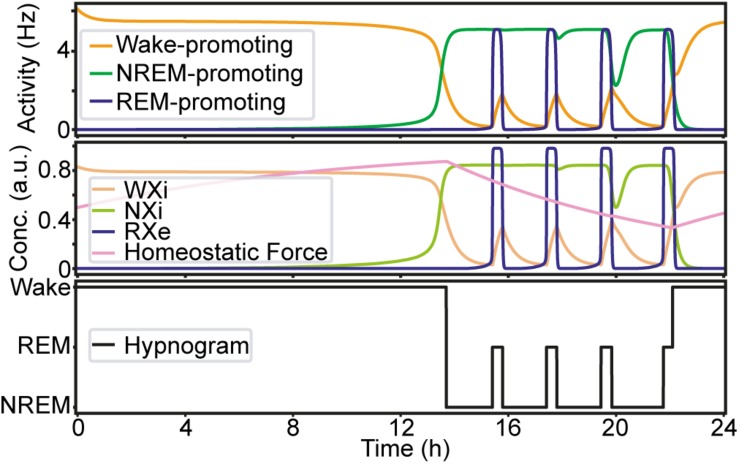
An example of the sleep–wake cycle generated by the network with the initial parameters. Top, the firing rate of each population as a function of time. Middle, the concentration of the neurotransmitters and the homeostatic force. Bottom, a hypnogram which was determined based on firing rates of the three neural populations.

Before manipulating synaptic weights across pathways, we confirmed the sleep–wake cycle in our model (Figure 2). The initial parameter setting in our model was the same as that in previous reports (Diniz Behn and Booth, 2012; Costa et al., 2016) (Supplementary Table S2). As shown in Figure 2, this network always started with wakefulness where activity in the Wake-promoting population was high. As the homeostatic force gradually built up, the Wake-promoting population dropped its activity and the network entered NREM sleep. During sleep, the homeostatic force gradually decreased while alternations between NREM sleep and REM sleep appeared before the network exhibited wakefulness again. As expected, the concentration of neurotransmitters was well correlated with the firing rate of neural populations.

**FIGURE 3:**
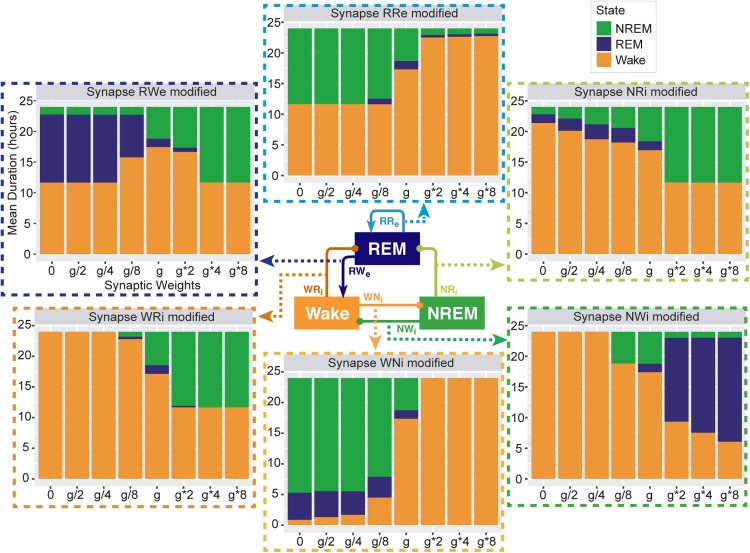
Total duration of each sleep state for different synaptic weights. Each bar graph represents the total duration of each state as a function of synaptic weights. The variable g represents the synaptic weight for the control condition. Each value is an average duration of each state from 8 simulations.

**FIGURE 4:**
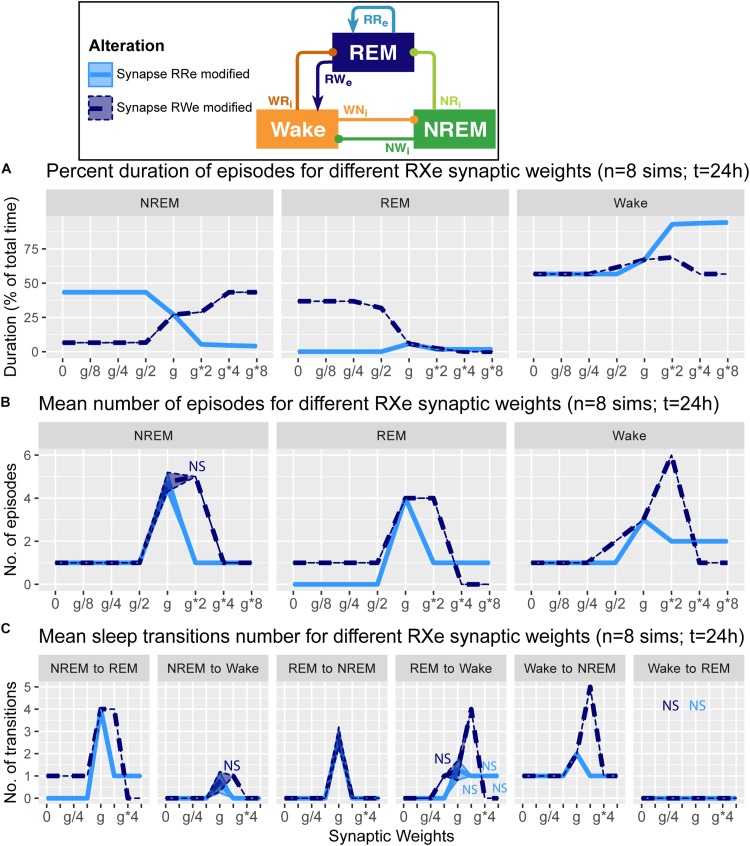
Effects of synaptic weight alterations of the REM population on sleep architecture. The percentage of time spent in each state **(A)**, the number of episodes **(B)**, and the number of state transitions **(C)** as a function of synaptic weights. Each profile was based on eight 24 h simulations. Data presents mean ± s.e.m. Light blue, RRe pathway; dark blue, RWe pathway. NS, non-significant (one-way ANOVA).

**FIGURE 5:**
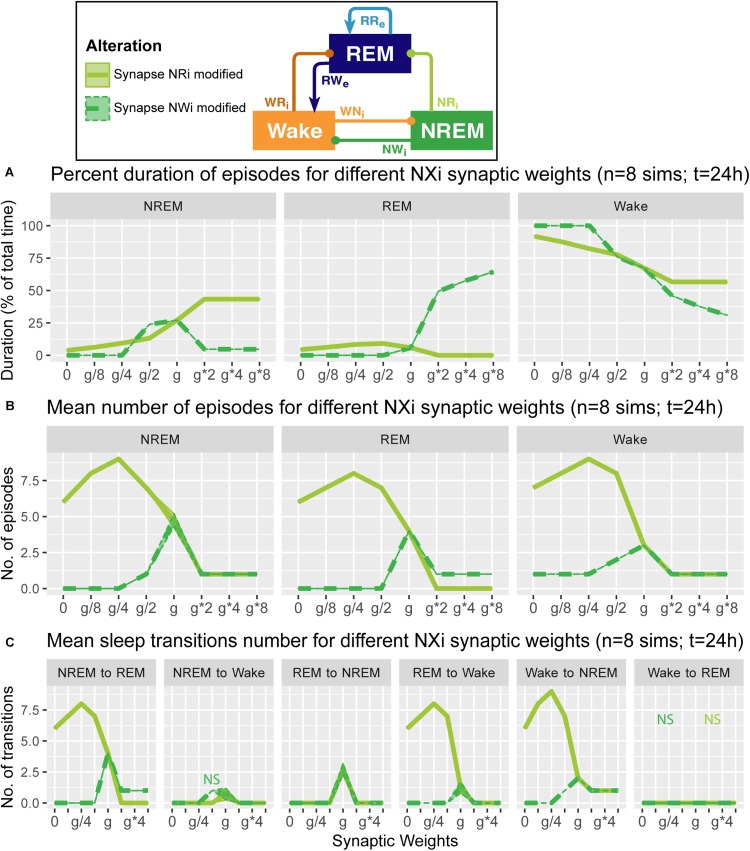
Effects of synaptic weight alterations of the NREM population on sleep architecture. The percentage of time spent in each state **(A)**, the number of episodes **(B)**, and the number of state transitions **(C)** as a function of synaptic weights. Each profile was based on eight 24 h simulations. Data presents mean ± s.e.m. Light green, NRi pathway; green, NWi pathway. NS, non-significant (one-way ANOVA).

**FIGURE 6:**
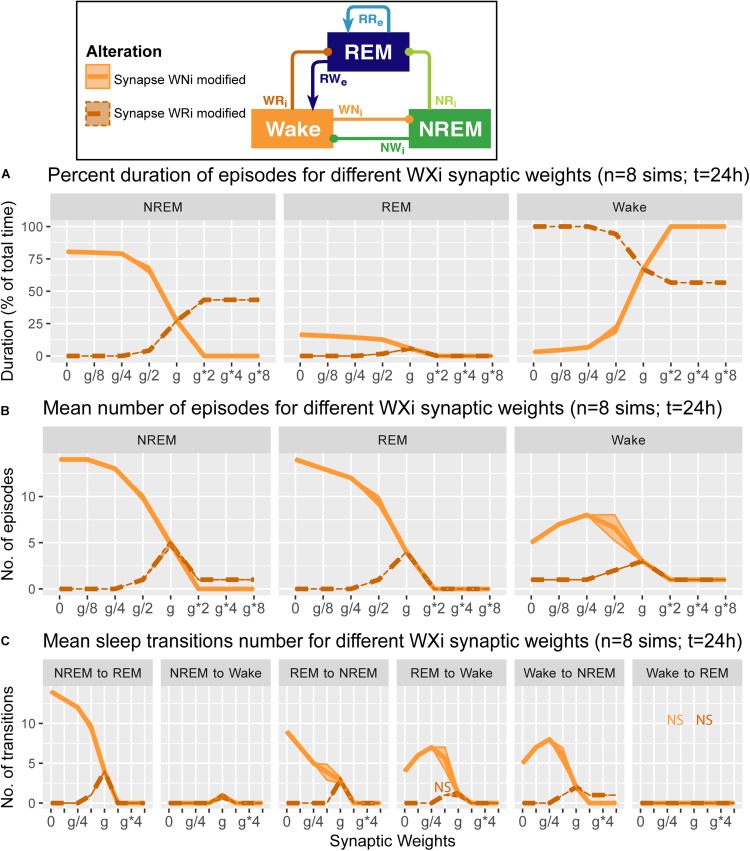
Effects of synaptic weight alterations of the Wake population on sleep architecture. The percentage of time spent in each state **(A)**, the number of episodes **(B)**, and the number of state transitions **(C)** as a function of synaptic weights. Each profile was based on eight 24 h simulations. Data presents mean ± s.e.m. Orange, WNi pathway; brown, WRi pathway. NS, non-significant (one-way ANOVA).

**FIGURE 7:**
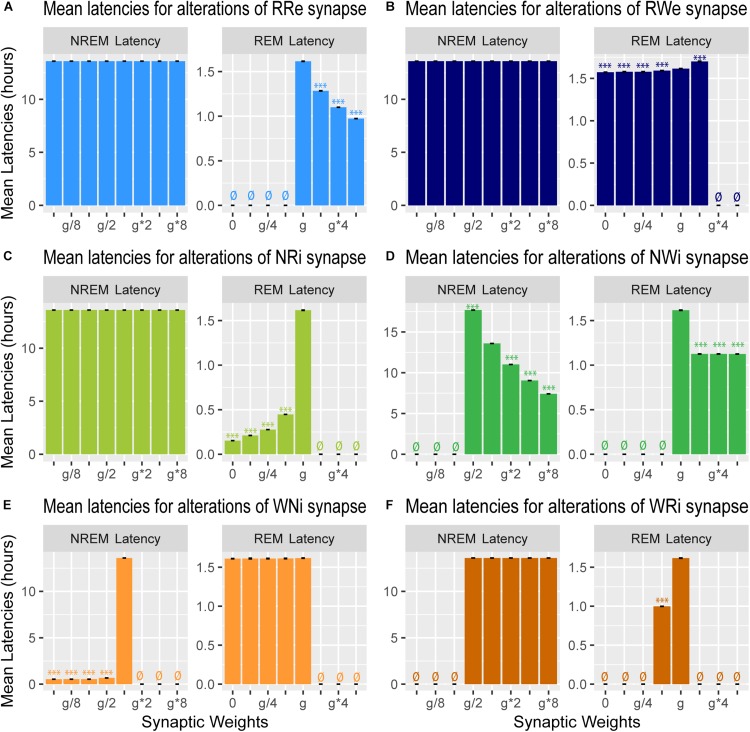
Effects of synaptic weight alterations on sleep latency. Bar graphs represent mean latency for NREM (left) and REM (right) as a function of synaptic weights in modifications of RRe **(A)**, RWe **(B)**, NRi **(C)**, NWi **(D)**, WNi **(E)**, and WRi pathways **(F)**. Error bars, s.e.m.; ø, no occurrence of the state.

In the following sections, to assess the effect of synaptic weight alterations on sleep architecture, we measured the following quantities, all of which are measurable experimentally:

• the total duration of each state (Figure 3 and Supplementary Figure S2),
• the percentage of the time spent in these states (Figures 4A, 5A, 6A),
• the number of episodes (Figures 4B, 5B, 6B),
• the number of transitions between states (Figures 4C, 5C, 6C), and
• the NREM and REM sleep latencies (Figure 7).

In the following sections, we describe how synaptic weight alterations affect sleep architecture in this network with respect to these measurements.

### Effects of Synaptic Weight Alteration on Total Sleep–Wake Duration

To investigate pathway-dependent regulation of sleep, we systematically modified the synaptic weight across pathways: the modified weight span from 0 to 8 times while *g* was the initial condition. We performed 24-h simulations (*n* = 8) in each condition.

To assess the overall sleep architecture, we measured the total duration of each state (Figure 3). While each neural population had two output pathways (Figure 1), the effect of weight alterations on sleep architecture was highly pathway-dependent: in the case of the outputs from the Wake population, although stronger weights in the Wake → NREM (WNi) pathway led to longer wakefulness (*F*_1_,_7_ = 911.4, *p* < 0.0001, one-way ANOVA), the Wake → REM (WRi) pathway showed an opposite trend (*F*_1_,_7_ = 88.7, *p* < 0.0001, one-way ANOVA). The WNi pathway was necessary to induce Wake whereas the WRi pathway was necessary to induce sleep states.

In the outputs from the NREM populations, stronger weights in the NREM → REM (NRi) connection led to longer NREM (*F*_1_,_7_ = 14985.8, *p* < 0.0001, one-way ANOVA) whereas stronger weights in the NREM → Wake (NWi) connection were associated with longer REM (*F*_1_,_7_ = 2290812, *p* < 0.0001, one-way ANOVA).

In the outputs from the REM population, to our surprise, strong recurrent excitatory (RRe) connection shortened the duration of REM sleep (*F*_1_,_7_ = 189.2, *p* < 0.0001, one-way ANOVA). Rather, weaker weighting in the REM → Wake (RWe) connection promoted longer REM sleep (*F*_1_,_7_ = 94156.8, *p* < 0.0001, one-way ANOVA). Thus, the effects of synaptic weight alterations on overall sleep architecture were highly pathway-dependent. We also assessed how simultaneous alterations of two output pathways from each neural population affect sleep dynamics (Supplementary Figure S3) (see below Section “Joint Alterations of Two Output Pathways From Each Population and Sleep Architecture” for comprehensive simultaneous alterations). The outcomes deviated from those of individual pathway manipulations, suggesting pathway-dependent regulation in the sleep dynamics. In the next sections, we explore detailed sleep architecture of this model with varied synaptic weights.

### Alterations of REM Population Output Pathways and Overall Sleep Architecture

How does the output from the REM population contribute in the sleep architecture? To address this, we quantified the effect of synaptic weight alterations in the REM population outputs on the sleep architecture, with respect to the percentage of time spent in each state (Figure 4A), the number of episodes (Figure 4B), and the number of state transitions (Figure 4C).

When we manipulated the synaptic weight in the RRe pathway (light blue in Figure 4), the percentage of NREM sleep decreased as a function of the synaptic weight (*F*_1_,_7_ = 1.93e5, *p* < 0.0001, one-way ANOVA) whereas the percentage of Wake increased (*F*_1_,_7_ = 8.63e5, *p* < 0.0001, one-way ANOVA) (Figure 4A). We observed only small changes in the percentage of REM sleep. The number of all episodes were generally reduced (Figure 4B): it was similar for NREM sleep no matter the synaptic weights (*F*_1_,_7_ = 4.78e2, *p* < 0.0001, one-way ANOVA), but we observed a smaller reduction in REM sleep and Wake episodes for stronger weights (*F*_1_,_7_ = 5.6 and *F*_1_,_7_ = 5.4 respectively, *p* < 0.0001 for both, one-way ANOVA). These results correlated with a similar reduction in the number of transitions between the states (Figure 4C). Thus, the manipulation of the RRe pathway stabilized the network state.

When we manipulated the synaptic weight in the RWe pathway (dark blue in Figure 4), the percentage of REM sleep decreased as a function of the synaptic weight (*F*_1_,_7_ = 9.31e5, *p* < 0.0001, one-way ANOVA) whereas the percentage of NREM sleep increased (*F*_1_,_7_ = 1.26e5, *p* < 0.0001, one-way ANOVA) (Figure 4A). The weaker weight in the RWe pathway extended the duration of REM sleep (*F*_1_,_7_ = 9.31e5, *p* < 0.0001, one-way ANOVA). Although the time spent in REM sleep decreased with *g*^∗^2 (*F*_1_,_7_ = 9.31e5, *p* < 0.0001, one-way ANOVA with *post hoc* Tukey HSD test), the number of REM episodes (*F*_1_,_7_ = 6.9, *p* < 0.0001, one-way ANOVA) (Figure 4B) and transitions (Figure 4C) increased. Hence stronger RWe pathway caused a fragmented sleep–wake cycle although *g*^∗^4 and *g*^∗^8 provided a different picture, suggesting an optimal range of synaptic strengths to induce the fragmentation of the sleep–wake cycle. Therefore, effects of alterations of REM population output pathways on sleep architecture were highly pathway-dependent.

### Alterations of NREM Population Output Pathways and Sleep Architecture

What are the effects of variation in the outputs from the NREM population in the sleep architecture and genesis? Here, we also examined how alterations of the output strengths from the NREM population contributed to sleep/wake transition, with respect to the percentage of time spent in each state (Figure 5A), the number of episodes (Figure 5B), and the number of state transitions (Figure 5C).

Strengthening the NRi pathway (light green in Figure 5) increased the percentage of time spent in NREM (*F*_1_,_7_ = 6.93e5, *p* < 0.0001, one-way ANOVA) and decreased that in REM (*F*_1_,_7_ = 4.62e5, *p* < 0.0001, one-way ANOVA) and Wake (*F*_1_,_7_ = 7.67e5, *p* < 0.0001, one-way ANOVA) (Figure 5A). This was associated with the reduction in state transitions (Figures 5B,C), meaning state stabilization. On the other hand, weakening the pathway increased the number of sleep episodes (*F*_1_,_7_ = 9.20e2, *p* < 0.0001, one-way ANOVA) and transitions (Figures 5B,C), meaning fragmentation.

Strengthening the NWi pathway (green in Figure 5) increased the percentage of time spent in REM sleep (*F*_1_,_7_ = 7.13e5, *p* < 0.0001, one-way ANOVA) and decreased that in NREM (*F*_1_,_7_ = 4.88e5, *p* < 0.0001, one-way ANOVA) and Wake (*F*_1_,_7_ = 7.37e5, *p* < 0.0001, one-way ANOVA) (Figure 5A). Weakening this pathway eliminated sleep episodes completely, meaning that this pathway is essential for sleep genesis.

### Alterations of Wake Population Output Pathways and Sleep Architecture

We also examined how alterations of the output strengths from the Wake population contributed to sleep architecture, with respect to the percentage of time spent in each state (Figure 6A), the number of episodes (Figure 6B), and the number of state transitions (Figure 6C).

When we manipulated the synaptic weights in the WNi pathway (orange in Figure 6), the percentage of Wake increased as the synaptic weight increased (*F*_1_,_7_ = 1.34e4, *p* < 0.0001, one-way ANOVA) (Figure 6A). On the other hand, as the synaptic weight decreased, the more the number of episodes increased across three states (*F*_1_,_7_ = 9750.7 for REM, *F*_1_,_7_ = 8.12e3 for NREM, *F*_1_,_7_ = 3.14e2 for Wake, *p* < 0.0001 for all, one-way ANOVA) (Figure 6B), with longer sleep states (*F*_1_,_7_ = 1.41e4, *p* < 0.0001, one-way ANOVA) (Figure 6A).

Contrary to these, when we increased the synaptic weight in the WRi pathway (brown in Figure 6), the percentage of Wake decreased (*F*_1_,_7_ = 5.72e5, *p* < 0.0001, one-way ANOVA) (Figure 6A). There was an optimal range to increase the numbers of sleep episodes (*F*_1_,_7_ = 1.27e3, *p* < 0.0001, one-way ANOVA) (Figure 6B). Again, the effects of alterations of Wake population output pathways on sleep architecture were pathway-dependent.

### Effects of Synaptic Modifications on the Sleep Latency

We also measured the latency of NREM and REM (Figure 7): the former is the latency of the first NREM episode since the beginning of the simulation whereas the latter is the latency of the first REM episode since the onset of the first NREM episode.

Strengthening the RRe pathway decreased the REM latency (*F*_7_,_56_ = 7.22e5, *p* < 0.0001, one-way ANOVA) (Figure 7A) whereas strengthening the RWe pathway increased the REM latency at g^∗^2 (*F*_7_,_56_ = 1.11e5, *p* < 0.0001, one-way ANOVA with *post hoc* Tukey HSD test) (Figure 7B). As expected, we did not observe any effect on the NREM latency by the manipulation of either pathway (Figures 7A,B). Thus, the output pathways from the REM population contributed only to the REM latency.

Weakening the NRi pathway decreased the REM latency (*F*_7_,_56_ = 4.43e5, *p* < 0.0001, one-way ANOVA) whereas the NREM latency was not changed (Figure 7C). Strengthening the NWi pathway decreased the NREM latency (*F*_7_,_56_ = 9,63e7, *p* < 0.0001, one-way ANOVA) whereas the REM latency was also reduced and remained consistent across different weights (*F*_7_,_56_ = 5.33e5, *p* < 0.0001, one-way ANOVA) (Figure 7D). Thus, the output pathways from the NREM population exhibited complex contributions to the NREM and REM latencies depending on output pathways.

Finally, weakening the WNi pathway decreased the NREM latency (*F*_7_,_56_ = 1.53e8, *p* < 0.0001, one-way ANOVA) whereas the REM latency was not affected as long as sleep was induced (Figure 7E). While strengthening the WRi pathway did not affect the NREM latency, the REM latency increased at *g^∗^2* (*F*_7_,_56_ = 8.29e5, *p* < 0.0001, one-way ANOVA with *post hoc* Tukey HSD test). Thus, the output pathways from the Wake population contributed to the latency of sleep state which was directly influenced.

### Effects of Synaptic Modifications on the Dynamics of Population Activity

Investigating the effect of synaptic modifications on the sleep architecture (Figures 4–6) and sleep latency (Figure 7), we noticed at least two non-trivial responses of the system. First, the strength of the RRe pathway did not correlate with the duration of REM sleep (Figure 4). Second, the stronger NWi pathway led to longer REM sleep, rather than longer NREM sleep (Figure 5).

**FIGURE 8:**
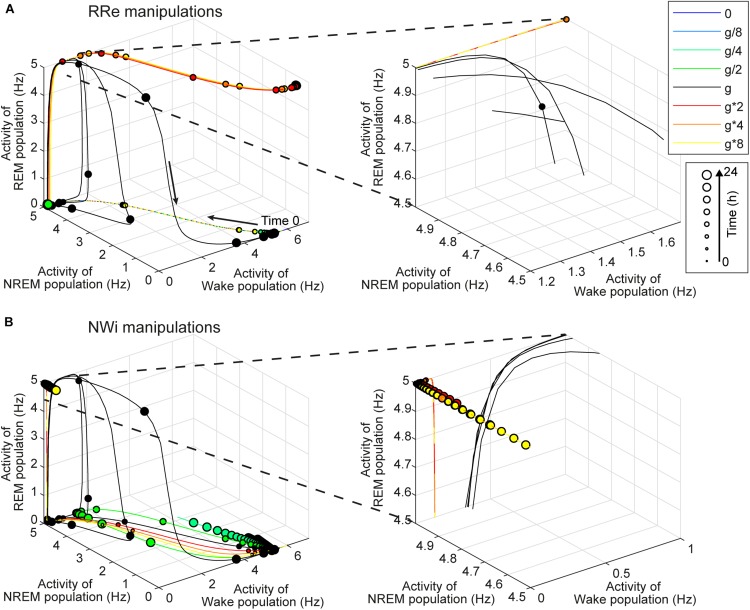
Effects of synaptic modifications on the dynamics of population activity. **(A)** Modifications of RRe pathway. **(B)** Modifications of NWi pathway. In each plot, the firing rate dynamics of three populations are shown in three-dimensional space. Line colors indicate types of synaptic modifications. Every 30-min time point is marked and their sizes represent time points of the simulation. Right panels show the magnified traces.

To gain insight into the underlying mechanism, we analyzed the firing rate dynamics of three populations (Figure 8). With respect to the manipulation of the RRe pathway (Figure 8A), in the default condition, the firing rate of the REM-promoting population quickly decreased. This was due to the inhibitory effect from the WRi pathway as the firing rate of the Wake-promoting population increased. However, when the strength of the RRe pathway increased, the firing rate of the REM-promoting population kept high along with increasing the firing rate of the Wake-promoting population. Therefore, by definition, the system entered and kept Wake. Thus, increasing the strength of the RRe pathway led to a pathological state where both the REM-promoting and Wake-promoting populations stay active.

With respect to the manipulation of the NWi pathway (Figure 8B), when the strength of the NWi pathway increased, the firing rate of the Wake-promoting population remained low and decreased due to the inhibitory effect of the NWi pathway. This resulted in the saturated firing rate of the REM-promoting population and therefore longer REM sleep. From these two analyses, an optimal range of activation in the Wake-promoting population plays a key role in the regulation of REM sleep.

### Joint Alterations of Two Output Pathways From Each Population and Sleep Architecture

**FIGURE 9:**
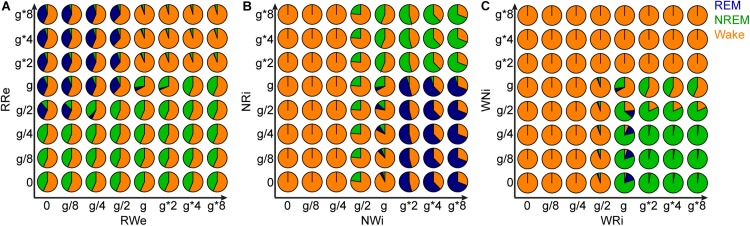
Effects of joint manipulation of two output pathways on the percentage of vigilance states. **(A)** The manipulation of output pathways from REM-promoting population. Each pie chart shows the percentage of three vigilance states at a certain joint manipulation. **(B)** The manipulation of output pathways from NREM-promoting population. **(C)** The manipulation of output pathways from Wake-promoting population.

Finally, to gain further insight into the role of each pathway in the behavior of this model, we manipulated the strength of the two output pathways from each population (Figure 9). Two types of joint manipulations could increase the total duration of REM sleep: first, the stronger RRe pathway with the weaker RWe pathway increased the duration of REM sleep (Figure 9A). This was consistent with the intuition obtained above (Figure 8A). Second, the weaker NRi pathway with the stronger NWi pathway also increased the duration of REM sleep (Figure 9B). To increase the total duration of NREM sleep, in addition to the weaker RRe pathway or stronger inhibitory synapses from the NREM-promoting population, the stronger WRi pathway with the weaker WNi pathway also lead to longer NREM sleep (Figure 9C). These results indicate that the balance between two outputs is crucial to determine the sleep architecture.

## Discussion

In this study, we have introduced a modeling framework to investigate the dynamics of the sleep–wake cycle and the effects of internal network manipulations (i.e., synaptic weight variations) on its regulation. We have implemented a simple computational model with three interconnected neural populations (Figure 1), each one promoting a different state of the sleep–wake cycle (wakefulness, REM and NREM sleep). We have comprehensively assessed how the manipulation of synaptic weight affects the dynamics of the sleep–wake cycle in our model. We found that effects of synaptic weight alterations on the sleep dynamics depend on the pathway where the synapse belongs (Figures 2–9). For example, the manipulation of the two outputs from the Wake-promoting population showed opposite outcomes: one was lengthening the wakefulness state whereas the other was shortening it. Thus, the sleep–wake dynamics is regulated in a pathway-dependent manner.

### Implications of the Current Study

In previous studies, the performances of network models have been explored (Diniz Behn and Booth, 2012; Diniz Behn et al., 2013; Weber, 2017) and these models can replicate sleep dynamics (Diniz-Behn and Booth, 2010) as well as state-dependent neural firing (Tamakawa et al., 2006). However, few studies have reported how the strength of synaptic connections between wake- and sleep-promoting populations contribute to the sleep architectures. The present or similar studies may lead to at least two directions: first, this type of simulations may provide insight into the underlying mechanisms of inter-species differences in sleep dynamics as well as pathological sleep conditions in humans. Second, given the advent of recent technological advance, such as optogenetics and chemogenetics, addressing this issue *in silico* may provide insight into the design of new experiments.

For example, the REM-promoting population in the current model presumably represents pontine cholinergic populations. Experimentally, the involvement of pontine cholinergic populations in the initiation and maintenance of REM sleep has been actively debated (Grace and Horner, 2015): lesion and pharmacological studies have provided inconsistent and contradictory results (Amatruda et al., 1975; Webster and Jones, 1988; Boissard et al., 2002; Grace et al., 2014). Even recent chemogenetic and optogenetic experiments also provided conflicting observations (Van Dort et al., 2015; Kroeger et al., 2017): chemogenetic activation has no effect on REM sleep whereas optogenetic activation can trigger REM sleep. Our observations (Figures 4, 8, 9) demonstrated that the activation of both pathways had little effect on REM sleep whereas a more specific manipulation can increase the duration of REM sleep (Figure 9). These results suggest that the complex balance of the synaptic strength between the RRe and RWe pathways may determine the duration of REM sleep. Therefore, it would be interesting to adopt pathway-specific manipulations of cholinergic activity to reconcile this issue in future.

Another intriguing observation is that measuring the latency of sleep states provided relatively intuitive outcomes. For example, strengthening the RRe pathway could reduce the REM latency without increasing the duration of REM sleep (Figure 7A), consistent with recent experimental observations (Carrera-Cañas et al., 2019). Strengthening the NWi pathways also reduced the NREM latency (Figure 7D). Thus, measuring the latency to change states may provide insights into the role of the manipulated pathway in sleep regulation.

Another general implication can be derived from the results that the pathways which are not directly connected to the REM population can contribute to the duration of REM sleep. It is possible that any manipulations can make distal impacts, resulting in unexpected state alternations. This effect is called “Diaschisis” or “shocked throughout,” describing the sudden loss of function in another portion of the brain through being linked with a distal, (directly) affected brain region (Carrera and Tononi, 2014; Otchy et al., 2015). This implies that experimental observations may need to be interpret with care. Our simulations directly demonstrated such indirect effects even in our simple model.

### Limitations and Possible Improvements

One of the major limitations in the present study is that the network model did not fully capture biological sleep-wake regulation. For example, the duration of REM sleep episodes typically increases during the sleep period. Our model did not implement such a homeostatic regulation of REM sleep. Therefore, some of our observations do not necessarily predict the behavior of biological circuits. To address these issues, it would be important to extend the network size to reflect more biological observations (Tamakawa et al., 2006). For example, the reciprocal interaction present in our model between Wake and REM promoting populations has been hypothesized to be a part of the REM sleep regulation, which can be under the control of a circadian modulation (Lu et al., 2006; Sapin et al., 2009; Costa et al., 2016). The model presented here does include an homeostatic sleep drive through the NREM-promoting population, but does not have any circadian modulation, which is known to be another important sleep drive (Fuller et al., 2006; Scammell et al., 2017; Weber et al., 2018; Herice et al., 2019). These effects could be implemented into the model by adding some corresponding populations such as the suprachiasmatic nucleus (SCN), which heavily influences the sleep/wake transitions (Fleshner et al., 2011; Booth and Diniz Behn, 2014).

In addition to the extension of the network, it would be interesting to refine the formalism of the model. Indeed, in this study we focused on the activity of the neural populations and network dynamics rather than on the activity of single neurons. Such a model with a more detailed formalism (with spiking neurons for example) would be attractive but its implementation would require more parameters derived from experimental work. More quantitative experimental data are certainly required to create even more realistic networks (Herice et al., 2019).

Another limitation to the present work is that we manipulated the synaptic strength during the entire simulation period. In biological experiments, however, manipulations can be transient, such as in optogenetic experiments (Adamantidis et al., 2007; Van Dort et al., 2015; Weber et al., 2015). It would be interesting to manipulate synaptic weights transiently in the network model.

Relating to this point, it may be also interesting to reconsider the definition of the state in the model. In particular, if the activity of each neural population is manipulated, the current definition (see section Materials and Methods) cannot be adopted because the activity of each population itself defines the state. To address this issue, it would be interesting to connect the modeled sleep–wake regulating circuit to cortical circuits and muscle units, through which the state of the system can be defined based on the activity of the cortical circuits or muscle units as in biological experiments. This direction will become an important topic to better understand how subcortical sleep-regulating circuits and cortical circuits interact with each other across the sleep–wake cycle and how recent closed-loop stimulation approaches affect neural circuit dynamics as well as connectivity (Marshall et al., 2006; Ngo et al., 2019).

## Conclusion

In conclusion, utilizing a simple network model of the sleep–wake cycle, we found pathway-dependent effects of synaptic weight manipulations on sleep architecture. Given the fact that even the simple network model can provide complex behaviors, designing *in vivo* experiments and interpreting the outcomes require careful considerations about the complexity of sleep–wake regulating circuits. A similar computational approach could complement to make specific predictions for *in vivo* experiments.

## Data Availability Statement

The datasets generated and analyzed for this study can be found on GitHub (https://github.com/Sakata-Lab/sleep-model) and at https://doi.org/10.15129/f3fe4727-a05e-469e-b762-6b2c8a9b5b5f.

## Author Contributions

SS and CH designed the project, performed the simulations, analyzed the data, and wrote the manuscript. CH developed the code.

## Conflict of Interest

The authors declare that the research was conducted in the absence of any commercial or financial relationships that could be construed as a potential conflict of interest.
