# Supplementary material for: Pathway-Dependent Regulation of Sleep Dynamics in a Network Model of the Sleep–Wake Cycle
Source: Front Neurosci. 2019 Dec 20;13:1380. doi: 10.3389/fnins.2019.01380 (PMC6933528; doi:10.3389/fnins.2019.01380)
Supplement: Supplementary file 1 [file Table_1.pdf]

# *Pathway-dependent regulation of sleep dynamics in a network model of sleep-wake cycle - Supplementary Material*

**Charlotte Héricé[1] and Shuzo Sakata[1*]**

[1] Strathclyde Institute of Pharmacy and Biomedical Sciences, University of Strathclyde, 161 Cathedral Street, Glasgow, G4 0RE, UK

*** Correspondence:**
Corresponding Author
shuzo.sakata@strath.ac.uk

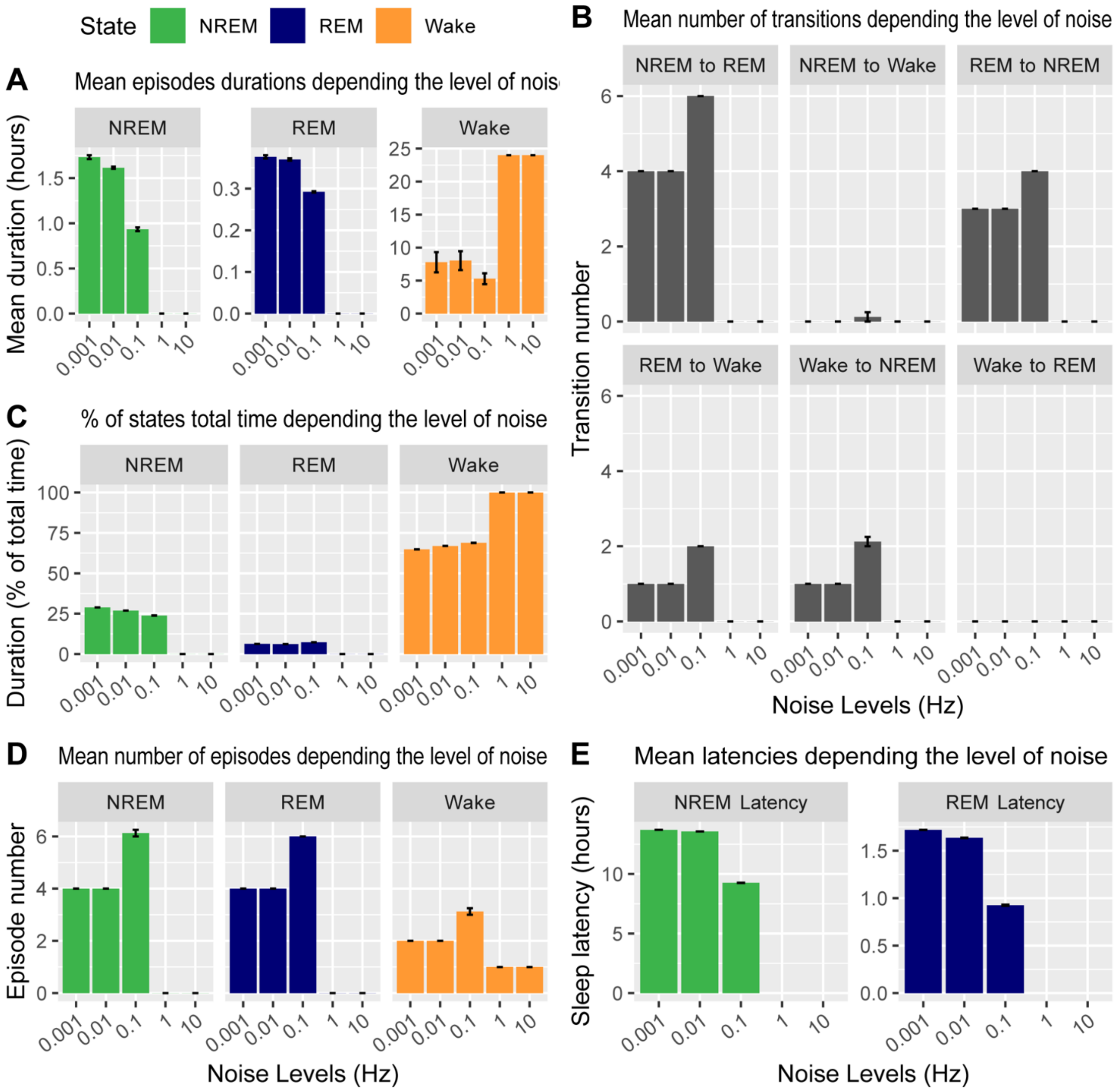


**Figure S1:** Effect of different levels of noise on the system dynamics. Different values of Gaussian noise (mean of 0.001Hz, 0.01Hz, 0.1Hz, 1Hz and 10Hz were applied during control conditions (n=8 simulations) and the mean duration of the episodes (A), the number of state transitions (B), the percentage of time spent in each state (C), the number of episodes and the sleep latencies were measured.

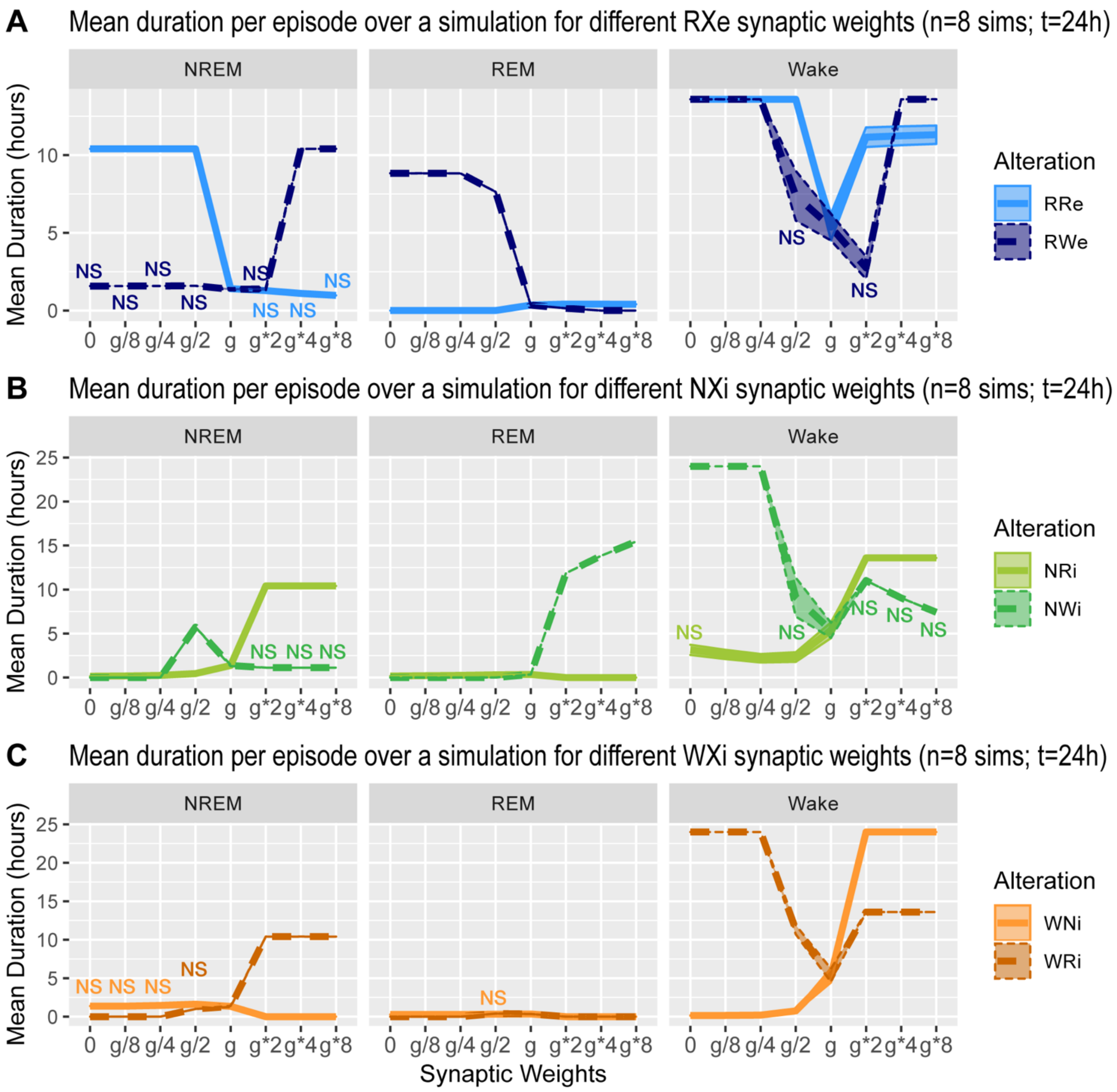


**Figure S2:** Mean episodes duration for different synaptic weights. The mean duration of each state as a function of synaptic weights for alterations in the REM (A), NREM (B) and (Wake) populations output pathways. Data presents mean ± s.e.m. NS, non-significant (one-way ANOVA).

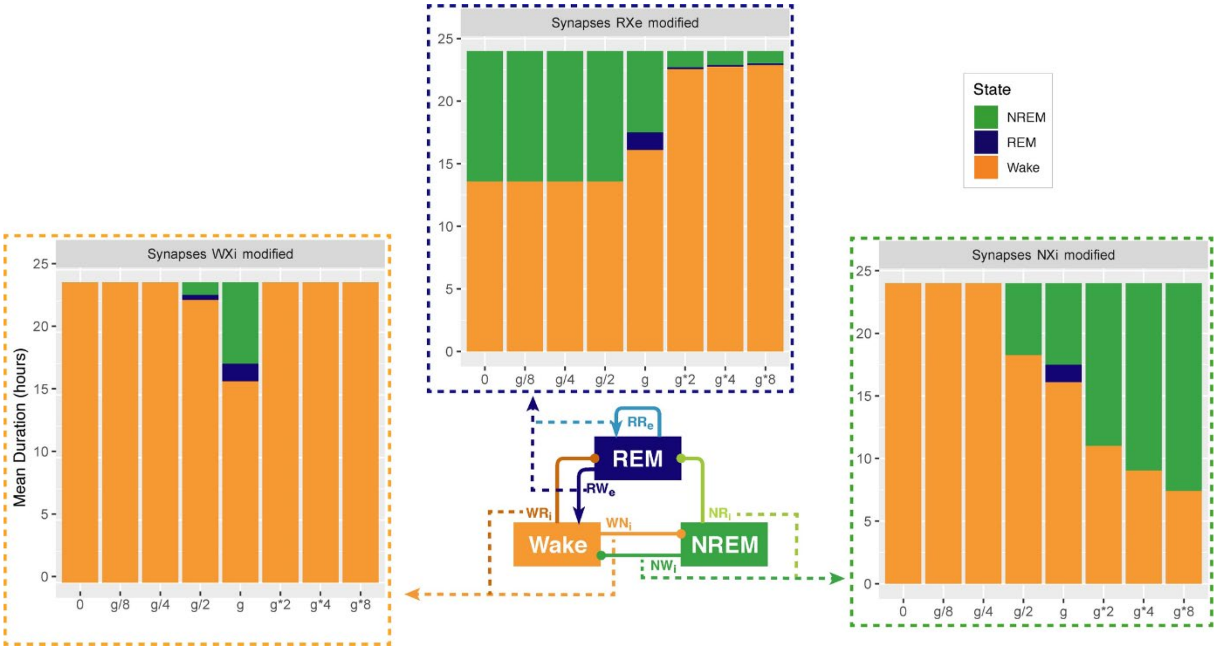


**Figure S3:** Total duration of each state for different synaptic weights and simultaneous alterations of two-output pathways. Each bar graph represents the total duration of each state as a function of synaptic weights. Here the synaptic alterations were made by two: RRe and RWe (top, blue arrows), NRi and NWi (right, green arrows), WNi and WRi (left, orange arrows) pathway. Each value is an average duration of each state from 8 simulation.

**Table S1:** Working environment parameters

| | |
|---|---|
| **OS** | Mac OS 10.14 (Mojave) |
| | Windows 10 |
| **Programming languages** | Python 3.6.8 (Anaconda installation) |
| | R for Mac OSX 3.5.1 (CRAN-R download) |
| | GNU Bash 3.2.57 |
| | MATLAB R2018b |
| **Libraries** | Numpy 1.13.1 (pip installation) |
| | Scipy 1.0.1 (pip installation) |
| | Matplotlib 2.2.2 (pip installation) |
| | Ggplot2 (CRAN-R download) |

**Table S2:** Parameters of the computational model. The label "See eq" refers to the equations in the Methods section.

| **Symbol** | **Value** | **Unit** | **Description** |
|---|---|---|---|
| $F_X$ | See eq | Hz | Firing rate of the population *X* |
| $F_{X\infty}$ | See eq | Hz | Steady-state firing rate for the population *X* |
| $I_X$ | See eq | / | Synaptic input function for the population *X* |
| $C_i$ | See eq | a.u. | Concentration of the neurotransmitter I |
| $C_{i\,\infty}$ | See eq | a.u. | Steady-state function for the release of the neurotransmitter I |
| $W_{max}$ | 6.5 | Hz | Maximum firing rate for the Wake-promoting population |

| | | | |
|---|---|---|---|
| $N_{max}$ | 5.0 | Hz | Maximum firing rate for the NREM-promoting population |
| $R_{max}$ | 5.0 | Hz | Maximum firing rate for the REM-promoting population |
| $\tau_W$ | $1500.10^3$ | ms | Membrane time constant of the Wake-promoting population |
| $\tau_N$ | $600.10^3$ | ms | Membrane time constant of the NREM-promoting population |
| $\tau_R$ | $60.10^3$ | ms | Membrane time constant of the REM-promoting population |
| $\tau_{RXe}$ | $10.10^3$ | ms | Membrane time constant of the neuromodulator RXe |
| $\tau_{NXi}$ | $10.10^3$ | ms | Membrane time constant of the neuromodulator NXi |
| $\tau_{WXi}$ | $25.10^3$ | ms | Membrane time constant of the neuromodulator WXi |
| $g_{RRe}$ | 1.6 | a.u. | Synaptic weight for the self-connection in the REM-promoting population |
| $g_{RWe}$ | 1.0 | a.u. | Synaptic weight for the connection from the REM- to the Wake-promoting populations |
| $g_{WNi}$ | -2.0 | a.u. | Synaptic weight for the connection from the Wake- to the NREM-promoting populations |
| $g_{WRi}$ | -4.0 | a.u. | Synaptic weight for the connection from the Wake- to the REM-promoting populations |
| $g_{NRi}$ | -1.3 | a.u. | Synaptic weight for the connection from the NREM- to the REM-promoting populations |
| $g_{NWi}$ | -1.68 | a.u. | Synaptic weight for the connection from the NREM- to the Wake-promoting populations |
| $\alpha_W$ | 0.5 | $ms^{-1}$ | Sigmoid slope parameter for the Wake-promoting population |
| $\alpha_N$ | 0.175 | $ms^{-1}$ | Sigmoid slope parameter for the NREM-promoting population |
| $\alpha_R$ | 0.13 | $ms^{-1}$ | Sigmoid slope parameter for the REM-promoting population |

| | | | |
|---|---|---|---|
| $\beta_W$ | -0.4 | $ms^{-1}$ | Sigmoid threshold parameters for the Wake-promoting population |
| $\beta_N$ | $- \kappa_N * h(t)$ | $ms^{-1}$ | Sigmoid threshold parameters for the NREM-promoting population |
| $\beta_R$ | -0.9 | $ms^{-1}$ | Sigmoid threshold parameters for the REM-promoting population |
| $\gamma_{RXe}$ | 2.0 | $ms^{-1}$ | Release scaling for neuromodulator RXe |
| $\gamma_{NXi}$ | 4.0 | $ms^{-1}$ | Release scaling for neuromodulator NXi |
| $\gamma_{WXi}$ | 5.0 | $ms^{-1}$ | Release scaling for neuromodulator WXi |
| $\kappa_N$ | -1.5 | a.u. | NREM-promoting population firing threshold modulation parameter |
| h(t) | See eq | a.u. | Homeostatic force |
| $H_{max}$ | 1.0 | a.u. | Maximum value for the homeostatic force |
| $\tau_{hw}$ | $34,830.10^3$ | ms | Time constant of sleep drive build up during wakefulness |
| $\tau_{hs}$ | $30,600.10^3$ | ms | Time constant of sleep drive decline during sleep |
| $\theta_W$ | 2.0 | $ms^{-1}$ | Sleep drive threshold |
